# Coronary atherosclerosis has a protective genetic causal effect against lung squamous cell carcinoma: A bidirectional two-sample Mendelian randomization study based on STROBE-MR guidelines

**DOI:** 10.1097/MD.0000000000043378

**Published:** 2025-07-25

**Authors:** Zhicheng Liao, Pengcheng Jia, Liang Pang, Yirui Chen, Jizhou Zhang

**Affiliations:** a Wenzhou TCM Hospital of Zhejiang Chinese Medical University, Wenzhou, Zhejiang Province, China.

**Keywords:** coronary atherosclerosis, lung cancer, lung squamous cell carcinoma, Mendelian randomization analysis, single-nucleotide polymorphism

## Abstract

Many observational studies have demonstrated an association between coronary atherosclerosis (CAS) and lung cancer (LUCA). However, there is not enough evidence to justify a direct genetic causal effect between CAS and LUCA as well as its subtypes. This study aimed to use Mendelian randomization (MR) analysis to assess the causal relationship between CAS and LUCA as well as its subtypes at the genetic level. This study was designed following the STROBE-MR guidelines and was a bidirectional two-sample MR analysis based on the context of the European population. It extracted genome-wide association study (GWAS) data of 51,589 CAS patients from the FinnGen Biobank database and extracted GWAS data of patients with LUCA and its subtypes from the IEU open GWAS database for MR analysis. In addition, this study used heterogeneity tests, sensitivity analyses, and multiple validity analyses to ensure the accuracy and robustness of the results. In the forward MR results, there was a statistically significant difference between CAS and LUCA (odds ratio [OR]: 0.88, 95% confidence interval [95% CI]: 0.82–0.95, *P* = .00099), and in the subtype analysis, there was a statistically significant difference between CAS and lung squamous cell carcinoma (LUSC) (OR [95% CI]: 0.84 [0.75–0.94], *P* = .00322); there was no statistical significance between CAS and either lung adenocarcinoma or small cell LUCA (OR [95% CI]: 0.93 [0.84–1.03], *P* = .1426; OR [95% CI]: 0.87 [0.73–1.03], *P* = .1145). In the reverse MR results, there was no statistical significance between LUCA and CAS as well as between LUSC and CAS (OR [95% CI]: 0.96 [0.92–1.01], *P* = .099; OR [95% CI]: 1.03 [0.95–1.10], *P* = .382). The study results showed that CAS has a genetic causal effect on LUCA and its subtype LUSC and that the effect is protective. Further studies are required to explore the underlying mechanisms.

## 1. Introduction

Lung cancer (LUCA) is the second most prevalent cancer and one of the cancers with a high mortality rate. LUCA can be divided into 2 main types: non-small cell lung cancer and small cell lung cancer (SCLC). Non-small cell lung cancer is the most common histologic type, accounting for 85% of all lung cancer cases. Non-small cell lung cancer is represented by lung adenocarcinoma (LUAD) and lung squamous cell carcinoma (LUSC).^[1]^

Atherosclerosis, the pathological basis of most cardiovascular diseases and the leading cause of cardiovascular morbidity and mortality worldwide, is characterized by the accumulation of lipids and inflammatory cells in the arterial wall.^[2]^ Coronary atherosclerosis (CAS) is a coronary atherosclerotic lesion whose development incorporates an arrangement of complex physiological and pathological processes, such as atherosclerotic plaque formation, plaque burst, and thrombosis, and eventually advances to coronary artery disease (CAD).^[3]^ According to data released by the World Health Organization, 17.7 million people die of cardiovascular diseases, which account for 31% of the global mortality rate, of which CAD patients account for approximately 7.4 million.^[4]^ Therefore, it is necessary to explore the initial pathological changes, namely CAS, in patients with CAD.

Although they are distinct diseases, recent research has revealed a link between CAS and LUCA. Sun et al found that CAD severity was associated with an increased risk of LUCA,^[5]^ while Liu et al showed that nearly 1/6 of deaths in CAD patients were caused by cancer, with cancer-specific mortality being highest for LUCA, and a meta-study revealed that CAD patients had a higher risk of developing LUCA.^[6,7]^ In contrast, a study by Swedish academics discovered that LUCA was associated with an increased risk of CAD,^[8]^ and multiple long-term follow-up studies have identified an increased risk of CAD in LUCA survivors.^[9,10]^ However, owing to the possible interference of potential confounders and the limitation of reverse causality bias, the observational study results do not directly illustrate the genetic level of causality between CAS and LUCA.

To investigate whether CAS is a risk factor for lung cancer, this study analyzed the causal effect of CAS as exposure on LUCA subtypes as outcome variables using Mendelian randomization (MR) methods, while the reverse causality was explored by interchanging the positions of the 2, which provided new ideas and strategies for the prevention and treatment of the disease.

## 2. Materials and methods

### 2.1. Study design

Since the alleles of individuals are randomly assigned and fixed at conception, MR analysis can circumvent the reverse causality and environmental confounders inherent in traditional epidemiological approaches.^[11]^ To avoid confounding factors due to ethnic differences, the European population with relatively complete genome-wide association study (GWAS) data was selected for this study. GWAS data of CAS patients were extracted from the FinnGen Biobank, while GWAS data of LUCA and its subtypes were extracted from the IEU open GWAS database. Single-nucleotide polymorphisms (SNPs) were used as IVs, and bidirectional MR analyses were performed to evaluate the causal associations between the IVs and outcome variables. Heterogeneity, sensitivity, and pleiotropy analyses were used to ensure the reliability and accuracy of the results.

**Figure 1.**
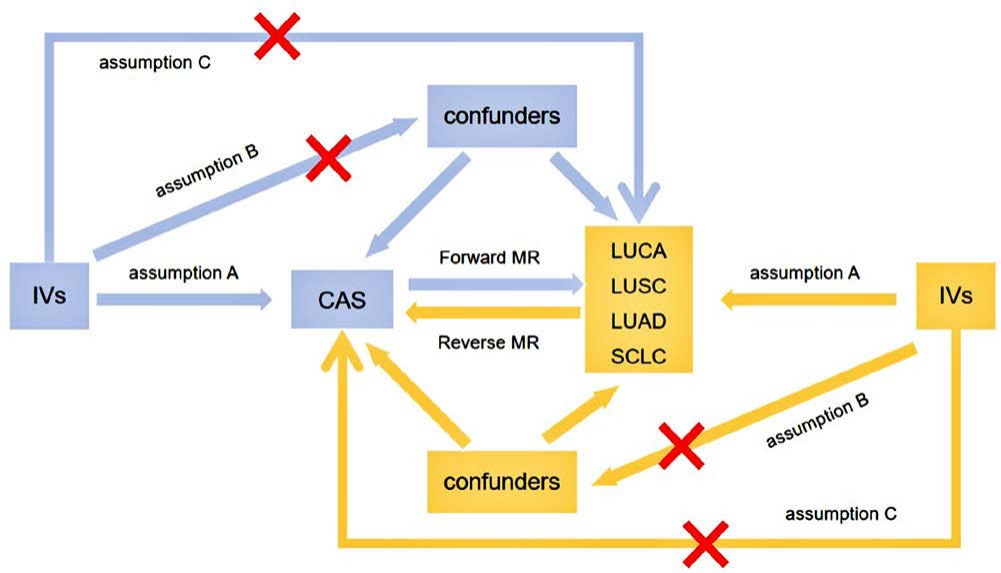
Three key assumptions of MR analysis. CAS = coronary atherosclerosis, IVs = instrumental variables, LUAD = lung adenocarcinoma, LUCA = lung cancer, LUSC = lung squamous cell carcinoma, MR = Mendelian randomization, SCLC = small cell lung cancer.

MR analysis is a research methodology based on 3 key assumptions,^[12]^ (A) there is a significant correlation between IVs and exposure factors (*P* < 5e-08); (B) IVs are not connected with any confounders of known exposure factor–outcome variable affiliations; and (C) IVs influence the outcome variables only through a significant association with the exposure factors (as shown in Fig. 1). This study was reported according to the STROBE-MR guidelines.^[13]^

### 2.2. Data sources

This study calculated the sample size using the “results” function in R software (power = 0.8, α = 0.05), and the result showed a sample estimate of 53,218 cases. So, we extracted the GWAS data for CAS patients from the FinnGen Biobank (51,589 samples and 343,079 controls). Meanwhile, we retrieved the GWAS summary data of LUCA and its subtypes from McKay study^[14]^ in the IEU open GWAS database using the following codes (ebi-a-GCST004748, ebi-a-GCST004744, ebi-a-GCST004750, and ebi-a-GCST004746), comprising 29,266 LUCA samples (11,273 LUAD, 7426 LUSC, and 2664 SCLC) and 56,450 controls. Specific information is provided in Table S1, Supplemental Digital Content, https://links.lww.com/MD/P432. According to the guidance received from our research ethics board, no ethics review is required for studies that use public datasets.

### 2.3. Instrumental variable (IV) selection

MR analysis is a method that uses genetic variation as an IV to test for causal relationships between exposure factors and diseases. SNPs selected as IVs were required to fulfill the following criteria: (1) SNPs must meet genome-wide significance criteria (*P* < 5e-08); (2) SNPs were free from linkage disequilibrium interference (parameter definitions: *r*^2^ < 0.001, kilobase pairs of 10,000 kbp); (3) to avoid weak instrumental bias from potentially overlapping datasets in MR analyses, all SNPs must ensure that their F-statistics are all >10, with the formula F = β^2^/SE^2^ (β stands for the effect value of the allele, and SE refers to the standard error of β); and (4) potentially pleiotropic SNPs have been removed by MR pleiotropy residual and MR_PRESSO_outlier methods. The selection of IVs was carried out using the R software (version 4.3.3).

In addition, smoking, radon exposure, air pollution, and lung diseases such as asthma and chronic obstructive pulmonary disease are the main risk factors for the advancement of LUCA,^[15]^ and these variables may bewilder the affiliation between CAS and LUCA. Therefore, to accommodate the assumption of independence (Assumption B), the LDtrait Tool was used to exclude SNPs associated with the above confounding factors. The final eligible SNPs are shown in Table S2, Supplemental Digital Content, https://links.lww.com/MD/P433.

### 2.4. MR analysis

In this study, bidirectional MR analyses were performed using the “TwoSampleMR” package in R 4.4.3, with inverse variance weighted (IVW) as the primary analysis method and with weighted median (WM) and MR Egger as the alternative analytical methods. In addition, if the IVs were greater than 3, the analysis was carried out using a random effects model; otherwise, a fixed effects model was used. The reliability of the MR analysis results is supported when the β-values of the MR Egger and WM methods are in a similar direction to the β-values of the IVW method. As multiple correlation tests were performed in the current study, Bonferroni calibration was adopted to reduce the risk of Type I errors, and the results were statistically significant only if the *P*-value < .05/6 = 0.00834. We applied the Cochran Q test to assess heterogeneity among individual genetic variance estimates, and a *P*-value < .05 suggests heterogeneity among SNPs and visualized the results by funnel plot. The MR estimates were calibrated by excluding potential outliers using the MR-PRESSO approach. The MR Egger intercept and MR-PRESSO global test were applied to assess potential horizontal pleiotropy. If either of the 2 results displayed a *P*-value < .05, it indicated the existence of horizontal pleiotropy, which suggests that IVs do not affect outcomes through exposure, violating the 3 main assumptions of the MR analysis. Demonstrating the association of each SNP with exposure–outcome using a scatter plot. In addition, each SNP was removed sequentially using the leave-one-out method to identify outliers that might affect the MR estimates, and the results were visualized in the form of a leave-one-out plot.

## 3. Results

### 3.1. Causal effects of CAS on LUCA and its subtypes

**Figure 2.**
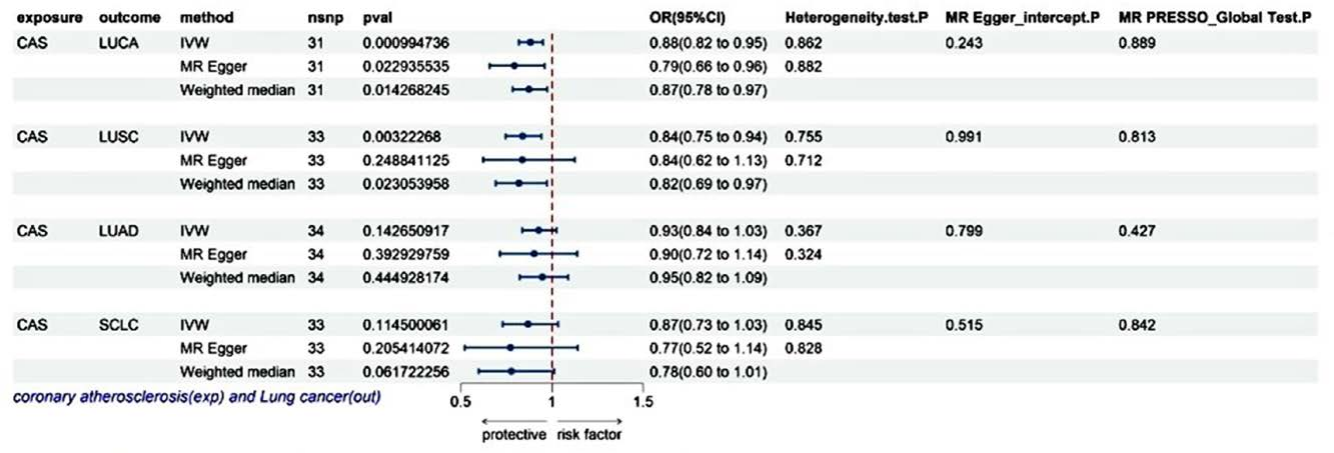
Causal effects of coronary atherosclerosis on lung cancer. CAS = coronary atherosclerosis, CI = confidence interval, exp = exposure, IVW = inverse variance weighted, LUAD = lung adenocarcinoma, LUCA = lung cancer, LUSC = lung squamous cell carcinoma, MR = Mendelian randomization, OR = odds ratio, out = outcome, SCLC = small cell lung cancer, SNP = single-nucleotide polymorphism.

The IVW results demonstrated a statistically significant difference in the effect of CAS on LUCA, with a risk odds ratio (OR) of 0.88 (95% confidence interval (CI), 0.82–0.95, *P* = .00099), and a statistically significant difference in the effect of CAS on LUSC (OR = 0.84, 95% CI: 0.75–0.94, *P* = .00322); whereas between CAS and LUAD, there was no statistical significance [OR (95% CI): 0.93 (0.84–1.03), *P* = .1426], also no statistical significance between CAS and SCLC [OR (95% CI): 0.87 (0.73–1.03), *P* = .1145] (as shown in Fig. 2).

In the heterogeneity analysis, the results reveal no heterogeneity between CAS and outcome variables (*P* > .05); in the pleiotropic inspections, the genetic causalities between exposure and outcome are unlikely to be affected by horizontal pleiotropy (*P*-values were >.05 for the MR Egger_intercept and MR PRESSO_Global tests), as shown in Figure 2; in the sensitivity analyses, the ORs values of MR Egger and WM methods were all <1, which was consistent with the ORs values of IVW, and after extracting the SNP one by one, the results showed that the effect of each IV was close to the overall effect, indicating that the MR analysis result was reliable and robust. Funnel, scatter, and leave-one-out plots are provided in Figure S3, Supplemental Digital Content, https://links.lww.com/MD/P434.

### 3.2. Causal effects of LUCA and its subtypes on CAS

**Figure 3.**
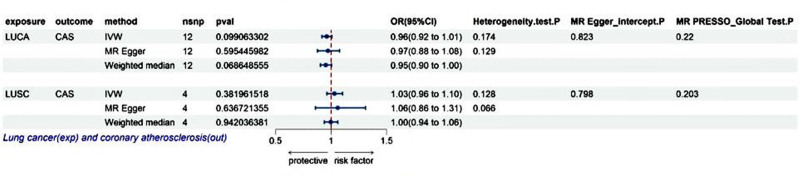
Causal effects of lung cancer on coronary atherosclerosis. CAS = coronary atherosclerosis, CI = confidence interval, exp = exposure, IVW = inverse variance weighted, LUCA = lung cancer, LUSC = lung squamous cell carcinoma, MR = Mendelian randomization, OR = odds ratio, out = outcome, SNP = single-nucleotide polymorphism.

After removing SNPs related to linkage disequilibrium, the number of SNPs in LUAD and SCLC did not support conducting reverse MR analysis for CAS in this study. The IVW results demonstrated no statistically significant difference in the effect of LUCA and its subtype LUSC on CAS [OR (95% CI): 0.96 (0.92–1.01), *P* = .099; OR (95% CI): 1.03 (0.95–1.10), *P* = .382], and the MR Egger and WM methods supported these IVW results. The results of heterogeneity tests, sensitivity analyses, and multiple validity analyses confirmed the reliability of the reverse MR analyses (as shown in Fig. 3). Funnel, scatter, and leave-one-out plots are provided in Figure S3, Supplemental Digital Content, https://links.lww.com/MD/P434.

## 4. Discussion

Previous prospective cohort study has found an association between CAD and LUCA, but no causal effect has been found between the 2 at the genetic level.^[16]^ Therefore, it is necessary and reasonable to use MR analysis to explore the genetic association between CAS and LUCA. In our study, the forward MR analyses revealed that CAS has a causal effect on LUCA and its subtype LUSC at the genetic level and that CAS is related to a decrease in the risk of LUCA and its subtype LUSC, whereas CAS does not have a genetically causal effect on LUAD and SCLC. The reverse MR analyses showed no causal effect of LUCA or LUSC on CAS at the genetic level. The results of the heterogeneity tests, multiple validity analyses, and sensitivity analyses prove that the bidirectional MR analysis results were reliable and robust. Based on our findings, we suggest that there may be a genetically protective effect of CAS on LUSC without interference from reverse causation, but it is not clear how the underlying mechanism functions. The following points were analyzed:

### 4.1. CAS may have a protective effect on LUCA and its subtype LUSC through the action of lipid metabolism markers

The genetic protective effect of CAS on LUCA and its subtype LUSC may be due to the action of lipid metabolism markers. The study showed that elevated levels of total cholesterol (TC) and low-density lipoprotein (LDL) are important factors in the occurrence and development of CAS,^[3]^ and elevated levels of TC and LDL are frequently detected in patients with CAS. Moreover, TC and LDL have been previously associated with cancer. It has been suggested that cholesterol secretes dendsaponin A, which induces tumor cell differentiation and death, and significantly suppresses tumor growth,^[17]^ at the same time, it has been suggested that high levels of LDL can down-regulate immunosuppression, decrease the activity or reactivity of the mevalonate pathway, and reduce the activity of the nuclear transcription factor NF-kB, thereby inhibiting the occurrence and development of cancer.^[18]^ For the association between TC and LUCA, multiple types of studies have found that TC levels are significantly related to a reduced risk of LUCA.^[19–21]^ Regarding the association between LDL and LUCA, an MR analysis of specific subtypes of LUCA which found that LDL levels were related to a reduced risk of LUSC rather than being significantly correlated with LUAD and SCLC, and our study which also found that CAS was associated with a reduced risk of LUSC and not significantly related to LUAD and SCLC, jointly suggest that LDL may play an important role in the relationship between CAS and LUSC.^[22]^ Although the mechanism of action of CAS on LUCA and its subtypes is unclear, we believe that higher TC and LDL levels in patients with CAS may play a role in reducing the risk of LUCA and its subtype LUSC based on our research findings.

### 4.2. CAS may have a protective effect on LUCA and its subtype LUSC through the action of the heart–gut–lung axis

#### 4.2.1. Heart–gut axis

Under the action of diet, inflammatory markers, hormones, gut microbiome, and environmental factors, the heart and gut have many complex interconnections, known as the “heart–gut axis.”^[23]^ Tuomisto and his team found several of the same bacteria detected in coronary artery plaques and intestinal feces in autopsy research on male patients and concluded that the bacteria in the plaques may have originated from the gut.^[24]^ Nakashima et al reported the discovery of several gut bacteria associated with the characterization of susceptible plaques in patients with cardiovascular diseases.^[25]^ The evidence from these studies fully supports the existence of a mechanism of interaction between CAS and the gut microbiota, which is the role of the heart–gut axis. Yu et al performed an observational study and found that the abundance of *Bacteroidetes* was lower in CAS patients than in healthy individuals^[26]^; Cui et al observed a decrease in the number of *Bacteroidetes* in patients with CAD^[27]^; Yoshida et al demonstrated the reductions of *Bacteroides Vulgatus* and *Bacteroides Dorie* in patients with CAD and suggested that these 2 organisms may suppress the higher systemic inflammatory response.^[28]^ In addition to changes in *Bacteroidetes*, recent studies have also reported, in patients with CAD, a reduction in the abundance of *Enterococcus*^[29]^ and an increase in the abundance of *Ruminococcus*.^[30]^ These findings imply that CAS plaques may act through the heart–gut axis during formation, leading to changes in gut microbiota.

#### 4.2.2. Gut–lung axis

An interaction exists between the gastrointestinal and respiratory tracts, referred to as the “gut–lung axis.” It has been suggested that the gut and lung interact through microbial and immune functions for bidirectional regulation, in which the gut microbiota activates B cells, T cells, and other immune cells that invade the lung through hematogenous and lymphatic pathways, activating pulmonary responses and inducing a variety of respiratory diseases.^[31,32]^ In recent years, many studies have also revealed that *Bacteroidetes*, *Enterococcus*, and *Ruminococcus* were related to LUCA and its subtypes. For *Bacteroidetes*, Liang et al found that it was strongly associated with lymph node metastasis of LUSC by constructing a microbiota-genome network,^[33]^ and Wei et al revealed that *Bacteroidetes* is correlated with an increase in NSCLC risk.^[34]^ For *Enterococcus*, Zhuang et al identified it as the highest potential biomarker for the development of LUCA.^[35]^ For *Ruminococcus*, an MR analysis study showed that *Ruminococcus_1* was a protective factor for LUCA and its subtype LUSC but was not significantly correlated with LUAD and SCLC,^[36]^ which is consistent with the pattern of causal associations in our findings.

Based on the results of our current study, we suggest that CAS may act through the heart–gut axis to cause the changes in the gut microbiota, which are represented by *Bacteroidetes*, *Enterococcus*, and *Ruminococcus*, thereby acting through the gut–lung axis to inhibit the development of LUCA and its subtype LUSC.

## 5. Limitations

This study has certain limitations as it did not conduct a more detailed stratified analysis of the exposure factors, such as stratifying by age and gender. The European population was selected as the genetic background for this study, which has some limitations in terms of ethnic coverage, and in the future, we will also further explore the association between CAS and LUCA and its subtypes in a multi-ethnic context. In addition, because of the limited number of SNPs, it is unclear whether CAS has a reverse genetic causal effect on LUAD and SCLC, more samples are necessary to support this study in the future.

## 6. Conclusion

In summary, the results of this study reveal a significant genetic protection causal effect of CAS on LUCA and its subtype LUSC, and no significant causal effect of CAS on LUAD and SCLC. In the reverse MR analysis, we found that there is no genetic causal effect of LUCA and LUSC on CAS. The exploration of the genetic causal relationship between CAS and LUCA with its subtypes in this study lays the basic foundation for subsequent deeper studies between the 2, provides new perspectives for elucidating the relationship between cardiovascular diseases and LUCA, and provides new ideas for the prevention and treatment of the diseases. In the future, we will continue to explore the mediating role of lipid markers and gut microbiota in the CAS-LUCA association, especially LDL and *Ruminococcus_1*.

## Acknowledgments

We express our gratitude for the valuable data resources provided by the researchers and the databases. This work was funded by the Natural Science Foundation of Zhejiang Province (project number LY20H290001) and by Zhejiang Chinese Medical University Special Research Projects of Affiliated Hospitals (project number 2022FSYYZZ29). The study funders/sponsors had no role in the design and conduct of the study.

## Author contributions

**Conceptualization:** Zhicheng Liao, Pengcheng Jia.

**Data curation:** Zhicheng Liao, Pengcheng Jia.

**Formal analysis:** Zhicheng Liao, Pengcheng Jia.

**Funding acquisition:** Zhicheng Liao, Jizhou Zhang.

**Methodology:** Zhicheng Liao, Pengcheng Jia.

**Resources:** Pengcheng Jia.

**Software:** Zhicheng Liao.

**Supervision:** Liang Pang, Yirui Chen.

**Validation:** Liang Pang, Yirui Chen.

**Visualization:** Yirui Chen.

**Writing – original draft:** Zhicheng Liao.

**Writing – review & editing:** Jizhou Zhang.

## Supplementary Material
