# Supplementary material for: Coronary atherosclerosis has a protective genetic causal effect against lung squamous cell carcinoma: A bidirectional two-sample Mendelian randomization study based on STROBE-MR guidelines
Source: Medicine (Baltimore). 2025 Jul 25;104(30):e43378. doi: 10.1097/MD.0000000000043378 (PMC12303480; doi:10.1097/MD.0000000000043378)
Supplement: Supplementary file 1 [file medi-104-e43378-s001.docx]

**Title**: Coronary atherosclerosis has a protective genetic causal effect against lung squamous cell carcinoma: a bidirectional two-sample Mendelian randomization study.

**First author**: Zhicheng liao

| Phenotype | Web source | N controls | N cases | Population | PMID | Year |
| --- | --- | --- | --- | --- | --- | --- |
| CAS | The FinnGen Biobank  (https://storage.googleapis.com/finngen-public-data-r10/summary_stats/finngen_R10_I9_CORATHER.gz) | 343,079 | 51,589 | European | NA | 2021 |
| LUCA | IEU open GWAS project: ebi-a-GCST004748  (https://gwas.mrcieu.ac.uk/datasets/ebi-a-GCST004748/) | 56,450 | 29,266 | European | 28604730 | 2017 |
| LUAD | IEU open GWAS project: ebi-a-GCST004744  (https://gwas.mrcieu.ac.uk/datasets/ebi-a-GCST004744/) | 55,483 | 11,273 | European | 28604730 | 2017 |
| LUSC | IEU open GWAS project: ebi-a-GCST004750  (https://gwas.mrcieu.ac.uk/datasets/ebi-a-GCST004750/) | 55,627 | 7,426 | European | 28604730 | 2017 |
| SCLC | IEU open GWAS project: GCST004746  (https://gwas.mrcieu.ac.uk/datasets/ebi-a-GCST004746/) | 21,444 | 2,664 | European | 28604730 | 2017 |

**Supplementary Material 1: Sample data sources used in this study and their specific information.**

CAS: coronary atherosclerosis, LUCA: lung cancer, LUAD: lung adenocarcinoma, LUSC: lung squamous cell carcinoma, SCLC: small cell lung carcinoma.
