# Supplementary material for: Coronary atherosclerosis has a protective genetic causal effect against lung squamous cell carcinoma: A bidirectional two-sample Mendelian randomization study based on STROBE-MR guidelines
Source: Medicine (Baltimore). 2025 Jul 25;104(30):e43378. doi: 10.1097/MD.0000000000043378 (PMC12303480; doi:10.1097/MD.0000000000043378)
Supplement: Supplementary file 2 [file medi-104-e43378-s002.docx]

**Title**: Coronary atherosclerosis has a protective genetic causal effect against lung squamous cell carcinoma: a bidirectional two-sample Mendelian randomization study.

**First author**: Zhicheng liao

**Supplementary Material 2:** **Instrumental variables for conducting MR analysis**

| CAS (exposure) and LUCA (outcome) | | | | | | | | | | | | | | | | | | | | | | | |
| --- | --- | --- | --- | --- | --- | --- | --- | --- | --- | --- | --- | --- | --- | --- | --- | --- | --- | --- | --- | --- | --- | --- | --- |
| SNP | effect_allele | other_allele | beta.exposure | | beta.outcome | eaf.exposure | | eaf.outcome | | chr | | pos | | se.outcome | | pval.outcome | | pval.exposure | | se.exposure | | F | |
| rs10514559 | C | G | -0.0475829 | | -0.003768 | 0.417284 | | 0.474721 | | 16 | | 82771255 | | 0.01182 | | 0.749915 | | 1.04E-09 | | 0.00779628 | | 37.25005839 | |
| rs10793514 | C | T | 0.0692693 | | 0.007866 | 0.694644 | | 0.662434 | | 10 | | 44496971 | | 0.012353 | | 0.524306 | | 1.56E-16 | | 0.00839426 | | 68.09524149 | |
| rs11591147 | T | G | -0.222579 | | -0.009328 | 0.0362264 | | 0.016124 | | 1 | | 55505647 | | 0.052975 | | 0.860225 | | 1.61E-25 | | 0.0213173 | | 109.0194482 | |
| rs11619038 | T | C | 0.0733329 | | 2.40E-05 | 0.118987 | | 0.12477 | | 13 | | 110923799 | | 0.017803 | | 0.998908 | | 5.19E-10 | | 0.0118027 | | 38.60423786 | |
| rs11970276 | G | A | 0.0531002 | | 0.000839 | 0.230917 | | 0.184992 | | 6 | | 2139450 | | 0.015326 | | 0.956322 | | 5.48E-09 | | 0.00910535 | | 34.00940431 | |
| rs12151108 | A | G | -0.136436 | | 0.018081 | 0.101158 | | 0.108664 | | 19 | | 11197261 | | 0.018423 | | 0.32639 | | 7.71E-26 | | 0.0129807 | | 110.4744202 | |
| rs12190287 | G | C | -0.0569984 | | 0.005981 | 0.447593 | | 0.369029 | | 6 | | 134214525 | | 0.012675 | | 0.637023 | | 1.83E-13 | | 0.00774356 | | 54.18062294 | |
| rs13139045 | C | T | -0.0537949 | | 0.008696 | 0.309757 | | 0.35146 | | 4 | | 120168588 | | 0.012219 | | 0.476654 | | 1.10E-10 | | 0.00833764 | | 41.62899538 | |
| rs137943060 | A | G | 0.245991 | | -0.033158 | 0.0186569 | | 0.034322 | | 14 | | 100080197 | | 0.043181 | | 0.44256 | | 1.15E-18 | | 0.0278928 | | 77.77754134 | |
| rs149076167 | C | A | 0.0813805 | | -0.015052 | 0.128554 | | 0.054505 | | 8 | | 22051281 | | 0.028015 | | 0.591065 | | 1.04E-12 | | 0.0114221 | | 50.76317665 | |
| rs2107595 | A | G | 0.0836528 | | -0.031769 | 0.193363 | | 0.170138 | | 7 | | 19049388 | | 0.015747 | | 0.0436405 | | 6.14E-18 | | 0.00969332 | | 74.47591782 | |
| rs2250645 | T | C | 0.0532786 | | 0.016797 | 0.350749 | | 0.301691 | | 10 | | 91008873 | | 0.013081 | | 0.199119 | | 3.85E-11 | | 0.00806048 | | 43.69017651 | |
| rs2291832 | A | G | 0.056624 | | -0.013483 | 0.745007 | | 0.701875 | | 1 | | 222826481 | | 0.014411 | | 0.349449 | | 1.58E-10 | | 0.0088507 | | 40.93038691 | |
| rs2395655 | G | A | -0.059378 | | 0.001999 | 0.362241 | | 0.395431 | | 6 | | 36645696 | | 0.012124 | | 0.869014 | | 1.67E-13 | | 0.00805396 | | 54.35408561 | |
| rs2900456 | C | T | 0.0438036 | | -0.004289 | 0.604334 | | 0.651823 | | 12 | | 20904468 | | 0.012154 | | 0.724192 | | 2.65E-08 | | 0.00787375 | | 30.94969448 | |
| rs3127580 | T | C | 0.0853116 | | -0.022799 | 0.123686 | | 0.127718 | | 6 | | 160710851 | | 0.017368 | | 0.189281 | | 1.33E-13 | | 0.0115231 | | 54.8122352 | |
| rs35081309 | A | T | 0.0787559 | | -0.015555 | 0.164982 | | 0.163164 | | 6 | | 133999756 | | 0.016141 | | 0.335196 | | 2.01E-14 | | 0.0102951 | | 58.5200999 | |
| rs3731204 | C | T | 0.0899224 | | -0.006901 | 0.103733 | | 0.128764 | | 9 | | 21987584 | | 0.017156 | | 0.687497 | | 6.05E-13 | | 0.0124905 | | 51.82939408 | |
| rs3752172 | A | G | -0.0594544 | | 0.024097 | 0.173167 | | 0.184835 | | 19 | | 42312933 | | 0.015251 | | 0.11411 | | 6.51E-09 | | 0.0102451 | | 33.67717066 | |
| rs3776299 | A | G | 0.0485479 | | -0.008651 | 0.505112 | | 0.471014 | | 5 | | 142507651 | | 0.011803 | | 0.46358 | | 3.16E-10 | | 0.00771723 | | 39.57473447 | |
| rs3777416 | G | A | -0.0522992 | | 0.004979 | 0.278012 | | 0.152331 | | 6 | | 160400196 | | 0.016405 | | 0.761502 | | 1.18E-09 | | 0.00859726 | | 37.00581467 | |
| rs380539 | T | G | 0.0529579 | | 0.004593 | 0.34197 | | 0.356188 | | 16 | | 86716058 | | 0.012426 | | 0.711672 | | 6.05E-11 | | 0.00809434 | | 42.80540641 | |
| rs4835377 | G | A | -0.0823538 | | -0.008698 | 0.778145 | | 0.8194 | | 4 | | 148039045 | | 0.015263 | | 0.56877 | | 3.98E-19 | | 0.00921447 | | 79.8778866 | |
| rs56225615 | T | C | 0.0970517 | | -0.027773 | 0.192771 | | 0.144784 | | 15 | | 79139370 | | 0.016798 | | 0.0982562 | | 9.62E-24 | | 0.00966127 | | 100.9108479 | |
| rs6750832 | G | A | 0.0594504 | | 0.016153 | 0.426348 | | 0.390253 | | 2 | | 85758090 | | 0.012207 | | 0.185729 | | 2.03E-14 | | 0.00777244 | | 58.50524546 | |
| rs7137258 | A | C | 0.0953264 | | -0.030296 | 0.0508112 | | 0.050198 | | 12 | | 54512164 | | 0.028637 | | 0.290101 | | 3.52E-08 | | 0.0172895 | | 30.39915533 | |
| rs72661887 | T | C | 0.0523715 | | -0.016689 | 0.537977 | | 0.442975 | | 1 | | 38416310 | | 0.012372 | | 0.177358 | | 1.11E-11 | | 0.00771087 | | 46.12997968 | |
| rs72710004 | T | C | 0.109539 | | -0.084154 | 0.0468209 | | 0.020552 | | 14 | | 100319598 | | 0.052505 | | 0.108986 | | 1.17E-09 | | 0.0180029 | | 37.02138018 | |
| rs7412 | T | C | -0.203095 | | 0.043928 | 0.0533155 | | 0.073124 | | 19 | | 45412079 | | 0.022366 | | 0.0495279 | | 3.97E-31 | | 0.0175033 | | 134.6351908 | |
| rs754203 | G | A | 0.0461634 | | -0.002139 | 0.294434 | | 0.311195 | | 14 | | 100157967 | | 0.012867 | | 0.867944 | | 4.28E-08 | | 0.0084259 | | 30.01670205 | |
| rs7696431 | G | T | -0.0456509 | | 0.003984 | 0.44007 | | 0.486467 | | 4 | | 169687725 | | 0.011878 | | 0.737322 | | 4.44E-09 | | 0.0077811 | | 34.42046194 | |
| rs9515203 | C | T | -0.0776043 | | 0.023449 | 0.2616 | | 0.25981 | | 13 | | 111049623 | | 0.014501 | | 0.105866 | | 9.21E-19 | | 0.00877451 | | 78.22144333 | |
| rs9852711 | T | C | -0.0792256 | | 0.01024 | 0.0827672 | | 0.023303 | | 3 | | 152151411 | | 0.040736 | | 0.801517 | | 2.14E-08 | | 0.0141477 | | 31.35879671 | |
|  |  |  |  | |  |  | |  | |  | |  | |  | |  | |  | |  | |  | |
| CAS(exposure) and LUAD(outcome) | | | | | | | | | | | | | | | | | | | | | | | |
| SNP | effect_allele | other_allele | beta.exposure | | beta.outcome | eaf.exposure | | eaf.outcome | | chr | | pos | | se.outcome | | pval.outcome | | pval.exposure | | se.exposure | | F | |
| rs10514559 | C | G | -0.0475829 | -0.011534 | | | 0.417284 | | 0.476643 | | 16 | | 82771255 | | 0.016367 | | 0.480999 | | 1.04E-09 | | 0.00779628 | | 37.25005839 |
| rs10793514 | C | T | 0.0692693 | 0.004234 | | | 0.694644 | | 0.661542 | | 10 | | 44496971 | | 0.017029 | | 0.803653 | | 1.56E-16 | | 0.00839426 | | 68.09524149 |
| rs11591147 | T | G | -0.222579 | 0.022676 | | | 0.0362264 | | 0.015857 | | 1 | | 55505647 | | 0.072364 | | 0.754006 | | 1.61E-25 | | 0.0213173 | | 109.0194482 |
| rs11619038 | T | C | 0.0733329 | -0.004971 | | | 0.118987 | | 0.125279 | | 13 | | 110923799 | | 0.024642 | | 0.840128 | | 5.19E-10 | | 0.0118027 | | 38.60423786 |
| rs117733303 | G | A | 0.400419 | 0.016763 | | | 0.0108855 | | 0.017686 | | 6 | | 160922870 | | 0.064639 | | 0.79538 | | 9.62E-30 | | 0.0353502 | | 128.3055973 |
| rs11970276 | G | A | 0.0531002 | 0.01038 | | | 0.230917 | | 0.183274 | | 6 | | 2139450 | | 0.021035 | | 0.621707 | | 5.48E-09 | | 0.00910535 | | 34.00940431 |
| rs12151108 | A | G | -0.136436 | -0.016545 | | | 0.101158 | | 0.106695 | | 19 | | 11197261 | | 0.025413 | | 0.515007 | | 7.71E-26 | | 0.0129807 | | 110.4744202 |
| rs12190287 | G | C | -0.0569984 | -0.004086 | | | 0.447593 | | 0.366734 | | 6 | | 134214525 | | 0.017315 | | 0.813462 | | 1.83E-13 | | 0.00774356 | | 54.18062294 |
| rs13139045 | C | T | -0.0537949 | -0.012427 | | | 0.309757 | | 0.35132 | | 4 | | 120168588 | | 0.016862 | | 0.461123 | | 1.10E-10 | | 0.00833764 | | 41.62899538 |
| rs149076167 | C | A | 0.0813805 | -0.005884 | | | 0.128554 | | 0.05549 | | 8 | | 22051281 | | 0.039388 | | 0.881255 | | 1.04E-12 | | 0.0114221 | | 50.76317665 |
| rs2107595 | A | G | 0.0836528 | -0.041522 | | | 0.193363 | | 0.16965 | | 7 | | 19049388 | | 0.021756 | | 0.0563184 | | 6.14E-18 | | 0.00969332 | | 74.47591782 |
| rs2250645 | T | C | 0.0532786 | 0.014965 | | | 0.350749 | | 0.303569 | | 10 | | 91008873 | | 0.018172 | | 0.410229 | | 3.85E-11 | | 0.00806048 | | 43.69017651 |
| rs2291832 | A | G | 0.056624 | -0.029621 | | | 0.745007 | | 0.699397 | | 1 | | 222826481 | | 0.019519 | | 0.129138 | | 1.58E-10 | | 0.0088507 | | 40.93038691 |
| rs2395655 | G | A | -0.059378 | -0.018917 | | | 0.362241 | | 0.391403 | | 6 | | 36645696 | | 0.016659 | | 0.256146 | | 1.67E-13 | | 0.00805396 | | 54.35408561 |
| rs2569550 | C | T | 0.0443148 | 0.02663 | | | 0.609894 | | 0.575365 | | 19 | | 11228745 | | 0.016353 | | 0.103432 | | 2.11E-08 | | 0.00790967 | | 31.38924414 |
| rs2900456 | C | T | 0.0438036 | -0.035132 | | | 0.604334 | | 0.657473 | | 12 | | 20904468 | | 0.016705 | | 0.0354552 | | 2.65E-08 | | 0.00787375 | | 30.94969448 |
| rs3005923 | A | G | -0.163015 | 0.006219 | | | 0.025974 | | 0.014344 | | 1 | | 56801542 | | 0.106093 | | 0.953255 | | 5.13E-11 | | 0.0248234 | | 43.12534731 |
| rs3127580 | T | C | 0.0853116 | -0.02074 | | | 0.123686 | | 0.12774 | | 6 | | 160710851 | | 0.024108 | | 0.38964 | | 1.33E-13 | | 0.0115231 | | 54.8122352 |
| rs35081309 | A | T | 0.0787559 | -0.034244 | | | 0.164982 | | 0.164791 | | 6 | | 133999756 | | 0.022245 | | 0.123702 | | 2.01E-14 | | 0.0102951 | | 58.5200999 |
| rs3731204 | C | T | 0.0899224 | 0.030391 | | | 0.103733 | | 0.128489 | | 9 | | 21987584 | | 0.023323 | | 0.19256 | | 6.05E-13 | | 0.0124905 | | 51.82939408 |
| rs3752172 | A | G | -0.0594544 | 0.038932 | | | 0.173167 | | 0.183715 | | 19 | | 42312933 | | 0.020935 | | 0.0629303 | | 6.51E-09 | | 0.0102451 | | 33.67717066 |
| rs3776299 | A | G | 0.0485479 | -0.01653 | | | 0.505112 | | 0.474432 | | 5 | | 142507651 | | 0.016393 | | 0.313264 | | 3.16E-10 | | 0.00771723 | | 39.57473447 |
| rs3777416 | G | A | -0.0522992 | -0.024261 | | | 0.278012 | | 0.149044 | | 6 | | 160400196 | | 0.023087 | | 0.293322 | | 1.18E-09 | | 0.00859726 | | 37.00581467 |
| rs380539 | T | G | 0.0529579 | -0.000558 | | | 0.34197 | | 0.3564 | | 16 | | 86716058 | | 0.017176 | | 0.974088 | | 6.05E-11 | | 0.00809434 | | 42.80540641 |
| rs4147997 | A | G | 0.0545727 | 0.004851 | | | 0.188253 | | 0.209797 | | 17 | | 66899290 | | 0.020014 | | 0.808471 | | 2.24E-08 | | 0.00975898 | | 31.27101849 |
| rs4835377 | G | A | -0.0823538 | -0.008193 | | | 0.778145 | | 0.821402 | | 4 | | 148039045 | | 0.021024 | | 0.696777 | | 3.98E-19 | | 0.00921447 | | 79.8778866 |
| rs56225615 | T | C | 0.0970517 | -0.014624 | | | 0.192771 | | 0.145778 | | 15 | | 79139370 | | 0.023093 | | 0.52655 | | 9.62E-24 | | 0.00966127 | | 100.9108479 |
| rs6750832 | G | A | 0.0594504 | -0.003011 | | | 0.426348 | | 0.386944 | | 2 | | 85758090 | | 0.016736 | | 0.85723 | | 2.03E-14 | | 0.00777244 | | 58.50524546 |
| rs7137258 | A | C | 0.0953264 | -0.057184 | | | 0.0508112 | | 0.049273 | | 12 | | 54512164 | | 0.038284 | | 0.135256 | | 3.52E-08 | | 0.0172895 | | 30.39915533 |
| rs72661887 | T | C | 0.0523715 | -0.007399 | | | 0.537977 | | 0.443619 | | 1 | | 38416310 | | 0.016938 | | 0.662229 | | 1.11E-11 | | 0.00771087 | | 46.12997968 |
| rs72710004 | T | C | 0.109539 | -0.139358 | | | 0.0468209 | | 0.020239 | | 14 | | 100319598 | | 0.072121 | | 0.0533237 | | 1.17E-09 | | 0.0180029 | | 37.02138018 |
| rs7412 | T | C | -0.203095 | 0.038146 | | | 0.0533155 | | 0.071471 | | 19 | | 45412079 | | 0.030804 | | 0.215583 | | 3.97E-31 | | 0.0175033 | | 134.6351908 |
| rs754203 | G | A | 0.0461634 | 0.009506 | | | 0.294434 | | 0.313154 | | 14 | | 100157967 | | 0.017756 | | 0.592405 | | 4.28E-08 | | 0.0084259 | | 30.01670205 |
| rs7696431 | G | T | -0.0456509 | 0.005364 | | | 0.44007 | | 0.488641 | | 4 | | 169687725 | | 0.016375 | | 0.743214 | | 4.44E-09 | | 0.0077811 | | 34.42046194 |
| rs9515203 | C | T | -0.0776043 | 0.026914 | | | 0.2616 | | 0.260216 | | 13 | | 111049623 | | 0.020028 | | 0.179009 | | 9.21E-19 | | 0.00877451 | | 78.22144333 |
| rs9852711 | T | C | -0.0792256 | 0.008738 | | | 0.0827672 | | 0.022145 | | 3 | | 152151411 | | 0.057443 | | 0.879096 | | 2.14E-08 | | 0.0141477 | | 31.35879671 |
|  |  |  |  |  | | |  | |  | |  | |  | |  | |  | |  | |  | |  |
| CAS(exposure) and LUSC(outcome) | | | | | | | | | | | | | | | | | | | | | | | |
| SNP | effect_allele | other_allele | beta.exposure | beta.outcome | | | eaf.exposure | | eaf.outcome | | chr | | pos | | se.outcome | | pval.outcome | | pval.exposure | | se.exposure | | F |
| rs10514559 | C | G | -0.0475829 | 0.011645 | | | 0.417284 | | 0.482427 | | 16 | | 82771255 | | 0.018943 | | 0.538706 | | 1.04E-09 | | 0.00779628 | | 37.25005839 |
| rs10793514 | C | T | 0.0692693 | 0.014819 | | | 0.694644 | | 0.663566 | | 10 | | 44496971 | | 0.019742 | | 0.452865 | | 1.56E-16 | | 0.00839426 | | 68.09524149 |
| rs11591147 | T | G | -0.222579 | -0.062446 | | | 0.0362264 | | 0.016151 | | 1 | | 55505647 | | 0.084285 | | 0.458758 | | 1.61E-25 | | 0.0213173 | | 109.0194482 |
| rs11619038 | T | C | 0.0733329 | 0.024376 | | | 0.118987 | | 0.125014 | | 13 | | 110923799 | | 0.028584 | | 0.393781 | | 5.19E-10 | | 0.0118027 | | 38.60423786 |
| rs117733303 | G | A | 0.400419 | -0.09722 | | | 0.0108855 | | 0.017733 | | 6 | | 160922870 | | 0.074733 | | 0.193296 | | 9.62E-30 | | 0.0353502 | | 128.3055973 |
| rs11970276 | G | A | 0.0531002 | -0.00136 | | | 0.230917 | | 0.183243 | | 6 | | 2139450 | | 0.024583 | | 0.955872 | | 5.48E-09 | | 0.00910535 | | 34.00940431 |
| rs12151108 | A | G | -0.136436 | 0.055853 | | | 0.101158 | | 0.105892 | | 19 | | 11197261 | | 0.029431 | | 0.0577231 | | 7.71E-26 | | 0.0129807 | | 110.4744202 |
| rs12190287 | G | C | -0.0569984 | 0.001163 | | | 0.447593 | | 0.366904 | | 6 | | 134214525 | | 0.020352 | | 0.954419 | | 1.83E-13 | | 0.00774356 | | 54.18062294 |
| rs149076167 | C | A | 0.0813805 | -0.071102 | | | 0.128554 | | 0.056943 | | 8 | | 22051281 | | 0.045214 | | 0.11582 | | 1.04E-12 | | 0.0114221 | | 50.76317665 |
| rs2107595 | A | G | 0.0836528 | -0.05664 | | | 0.193363 | | 0.168975 | | 7 | | 19049388 | | 0.025172 | | 0.0244399 | | 6.14E-18 | | 0.00969332 | | 74.47591782 |
| rs2250645 | T | C | 0.0532786 | 0.002356 | | | 0.350749 | | 0.305209 | | 10 | | 91008873 | | 0.021022 | | 0.910771 | | 3.85E-11 | | 0.00806048 | | 43.69017651 |
| rs2291832 | A | G | 0.056624 | 0.00159 | | | 0.745007 | | 0.699988 | | 1 | | 222826481 | | 0.023305 | | 0.945599 | | 1.58E-10 | | 0.0088507 | | 40.93038691 |
| rs2395655 | G | A | -0.059378 | 0.017901 | | | 0.362241 | | 0.389653 | | 6 | | 36645696 | | 0.019331 | | 0.354443 | | 1.67E-13 | | 0.00805396 | | 54.35408561 |
| rs2569550 | C | T | 0.0443148 | -0.001639 | | | 0.609894 | | 0.574118 | | 19 | | 11228745 | | 0.018927 | | 0.931 | | 2.11E-08 | | 0.00790967 | | 31.38924414 |
| rs2900456 | C | T | 0.0438036 | -0.003022 | | | 0.604334 | | 0.658637 | | 12 | | 20904468 | | 0.01936 | | 0.875979 | | 2.65E-08 | | 0.00787375 | | 30.94969448 |
| rs3127580 | T | C | 0.0853116 | -0.033192 | | | 0.123686 | | 0.127603 | | 6 | | 160710851 | | 0.027831 | | 0.233025 | | 1.33E-13 | | 0.0115231 | | 54.8122352 |
| rs35081309 | A | T | 0.0787559 | -0.00911 | | | 0.164982 | | 0.164235 | | 6 | | 133999756 | | 0.025888 | | 0.724918 | | 2.01E-14 | | 0.0102951 | | 58.5200999 |
| rs3731204 | C | T | 0.0899224 | -0.030054 | | | 0.103733 | | 0.126021 | | 9 | | 21987584 | | 0.027587 | | 0.275979 | | 6.05E-13 | | 0.0124905 | | 51.82939408 |
| rs3752172 | A | G | -0.0594544 | 0.029224 | | | 0.173167 | | 0.183117 | | 19 | | 42312933 | | 0.024449 | | 0.231962 | | 6.51E-09 | | 0.0102451 | | 33.67717066 |
| rs3776299 | A | G | 0.0485479 | -0.022116 | | | 0.505112 | | 0.475107 | | 5 | | 142507651 | | 0.018959 | | 0.243424 | | 3.16E-10 | | 0.00771723 | | 39.57473447 |
| rs3777416 | G | A | -0.0522992 | -0.001749 | | | 0.278012 | | 0.153232 | | 6 | | 160400196 | | 0.026072 | | 0.946529 | | 1.18E-09 | | 0.00859726 | | 37.00581467 |
| rs380539 | T | G | 0.0529579 | -0.000937 | | | 0.34197 | | 0.356336 | | 16 | | 86716058 | | 0.019951 | | 0.962527 | | 6.05E-11 | | 0.00809434 | | 42.80540641 |
| rs4835377 | G | A | -0.0823538 | -0.017972 | | | 0.778145 | | 0.82075 | | 4 | | 148039045 | | 0.024433 | | 0.462011 | | 3.98E-19 | | 0.00921447 | | 79.8778866 |
| rs553741 | C | G | 0.0640631 | 0.022233 | | | 0.637601 | | 0.63577 | | 1 | | 55520408 | | 0.020394 | | 0.275634 | | 1.69E-15 | | 0.00804601 | | 63.39496472 |
| rs56225615 | T | C | 0.0970517 | -0.026866 | | | 0.192771 | | 0.146819 | | 15 | | 79139370 | | 0.026888 | | 0.317707 | | 9.62E-24 | | 0.00966127 | | 100.9108479 |
| rs629301 | T | G | 0.0891313 | 0.006987 | | | 0.785458 | | 0.784143 | | 1 | | 109818306 | | 0.022357 | | 0.754637 | | 3.62E-21 | | 0.00943867 | | 89.1741292 |
| rs665770 | A | G | 0.0458422 | -0.042964 | | | 0.397902 | | 0.403733 | | 1 | | 201748124 | | 0.019216 | | 0.0253606 | | 4.97E-09 | | 0.00783894 | | 34.19922169 |
| rs6750832 | G | A | 0.0594504 | 0.001937 | | | 0.426348 | | 0.386024 | | 2 | | 85758090 | | 0.019531 | | 0.920993 | | 2.03E-14 | | 0.00777244 | | 58.50524546 |
| rs7137258 | A | C | 0.0953264 | -0.033725 | | | 0.0508112 | | 0.048625 | | 12 | | 54512164 | | 0.045489 | | 0.458453 | | 3.52E-08 | | 0.0172895 | | 30.39915533 |
| rs72661887 | T | C | 0.0523715 | -0.036298 | | | 0.537977 | | 0.446174 | | 1 | | 38416310 | | 0.019878 | | 0.0678516 | | 1.11E-11 | | 0.00771087 | | 46.12997968 |
| rs72710004 | T | C | 0.109539 | -0.055742 | | | 0.0468209 | | 0.020635 | | 14 | | 100319598 | | 0.082731 | | 0.500452 | | 1.17E-09 | | 0.0180029 | | 37.02138018 |
| rs754203 | G | A | 0.0461634 | -0.0145 | | | 0.294434 | | 0.313215 | | 14 | | 100157967 | | 0.020543 | | 0.480272 | | 4.28E-08 | | 0.0084259 | | 30.01670205 |
| rs7696431 | G | T | -0.0456509 | 0.017309 | | | 0.44007 | | 0.48633 | | 4 | | 169687725 | | 0.019051 | | 0.363567 | | 4.44E-09 | | 0.0077811 | | 34.42046194 |
| rs9515203 | C | T | -0.0776043 | -0.001593 | | | 0.2616 | | 0.260651 | | 13 | | 111049623 | | 0.023319 | | 0.945523 | | 9.21E-19 | | 0.00877451 | | 78.22144333 |
| rs9852711 | T | C | -0.0792256 | 0.033365 | | | 0.0827672 | | 0.022825 | | 3 | | 152151411 | | 0.06455 | | 0.605236 | | 2.14E-08 | | 0.0141477 | | 31.35879671 |
|  |  |  |  |  | | |  | |  | |  | |  | |  | |  | |  | |  | |  |
| CAS(exposure) and SCLC(outcome) | | | | | | | | | | | | | | | | | | | | | | | |
| SNP | effect_allele | other_allele | beta.exposure | beta.outcome | | | eaf.exposure | | eaf.outcome | | chr | | pos | | se.outcome | | pval.outcome | | pval.exposure | | se.exposure | | F |
| rs10514559 | C | G | -0.0475829 | 0.00234 | | | 0.417284 | | 0.461601 | | 16 | | 82771255 | | 0.029915 | | 0.93766 | | 1.04E-09 | | 0.00779628 | | 37.25005839 |
| rs10793514 | C | T | 0.0692693 | 0.023212 | | | 0.694644 | | 0.661613 | | 10 | | 44496971 | | 0.031284 | | 0.458107 | | 1.56E-16 | | 0.00839426 | | 68.09524149 |
| rs11591147 | T | G | -0.222579 | -0.061241 | | | 0.0362264 | | 0.016912 | | 1 | | 55505647 | | 0.128673 | | 0.634114 | | 1.61E-25 | | 0.0213173 | | 109.0194482 |
| rs11619038 | T | C | 0.0733329 | 0.046584 | | | 0.118987 | | 0.121931 | | 13 | | 110923799 | | 0.044472 | | 0.29487 | | 5.19E-10 | | 0.0118027 | | 38.60423786 |
| rs117733303 | G | A | 0.400419 | 0.123629 | | | 0.0108855 | | 0.017025 | | 6 | | 160922870 | | 0.123066 | | 0.315103 | | 9.62E-30 | | 0.0353502 | | 128.3055973 |
| rs12151108 | A | G | -0.136436 | 0.046085 | | | 0.101158 | | 0.113268 | | 19 | | 11197261 | | 0.045884 | | 0.315194 | | 7.71E-26 | | 0.0129807 | | 110.4744202 |
| rs12190287 | G | C | -0.0569984 | -0.028653 | | | 0.447593 | | 0.373846 | | 6 | | 134214525 | | 0.032008 | | 0.370699 | | 1.83E-13 | | 0.00774356 | | 54.18062294 |
| rs13139045 | C | T | -0.0537949 | 0.016938 | | | 0.309757 | | 0.349327 | | 4 | | 120168588 | | 0.030808 | | 0.582459 | | 1.10E-10 | | 0.00833764 | | 41.62899538 |
| rs137943060 | A | G | 0.245991 | -0.051969 | | | 0.0186569 | | 0.028389 | | 14 | | 100080197 | | 0.110554 | | 0.638299 | | 1.15E-18 | | 0.0278928 | | 77.77754134 |
| rs149076167 | C | A | 0.0813805 | 0.034248 | | | 0.128554 | | 0.048147 | | 8 | | 22051281 | | 0.069047 | | 0.619885 | | 1.04E-12 | | 0.0114221 | | 50.76317665 |
| rs2107595 | A | G | 0.0836528 | 0.024097 | | | 0.193363 | | 0.17761 | | 7 | | 19049388 | | 0.039563 | | 0.542473 | | 6.14E-18 | | 0.00969332 | | 74.47591782 |
| rs2291832 | A | G | 0.056624 | -0.020119 | | | 0.745007 | | 0.710932 | | 1 | | 222826481 | | 0.03654 | | 0.581904 | | 1.58E-10 | | 0.0088507 | | 40.93038691 |
| rs2395655 | G | A | -0.059378 | 0.027187 | | | 0.362241 | | 0.414605 | | 6 | | 36645696 | | 0.030514 | | 0.372945 | | 1.67E-13 | | 0.00805396 | | 54.35408561 |
| rs2569550 | C | T | 0.0443148 | -0.027058 | | | 0.609894 | | 0.573986 | | 19 | | 11228745 | | 0.029784 | | 0.363627 | | 2.11E-08 | | 0.00790967 | | 31.38924414 |
| rs2900456 | C | T | 0.0438036 | -0.001448 | | | 0.604334 | | 0.628164 | | 12 | | 20904468 | | 0.030474 | | 0.962104 | | 2.65E-08 | | 0.00787375 | | 30.94969448 |
| rs3127580 | T | C | 0.0853116 | -0.022952 | | | 0.123686 | | 0.128559 | | 6 | | 160710851 | | 0.043558 | | 0.598241 | | 1.33E-13 | | 0.0115231 | | 54.8122352 |
| rs35081309 | A | T | 0.0787559 | -0.05535 | | | 0.164982 | | 0.158777 | | 6 | | 133999756 | | 0.041642 | | 0.183787 | | 2.01E-14 | | 0.0102951 | | 58.5200999 |
| rs3731204 | C | T | 0.0899224 | -0.080671 | | | 0.103733 | | 0.132505 | | 9 | | 21987584 | | 0.043931 | | 0.0663117 | | 6.05E-13 | | 0.0124905 | | 51.82939408 |
| rs3752172 | A | G | -0.0594544 | -0.009744 | | | 0.173167 | | 0.188254 | | 19 | | 42312933 | | 0.038616 | | 0.800783 | | 6.51E-09 | | 0.0102451 | | 33.67717066 |
| rs3777416 | G | A | -0.0522992 | -0.001597 | | | 0.278012 | | 0.154943 | | 6 | | 160400196 | | 0.040949 | | 0.968896 | | 1.18E-09 | | 0.00859726 | | 37.00581467 |
| rs4147997 | A | G | 0.0545727 | 0.007659 | | | 0.188253 | | 0.20183 | | 17 | | 66899290 | | 0.03708 | | 0.836353 | | 2.24E-08 | | 0.00975898 | | 31.27101849 |
| rs4835377 | G | A | -0.0823538 | -0.016569 | | | 0.778145 | | 0.812211 | | 4 | | 148039045 | | 0.038214 | | 0.664593 | | 3.98E-19 | | 0.00921447 | | 79.8778866 |
| rs553741 | C | G | 0.0640631 | 0.004492 | | | 0.637601 | | 0.635008 | | 1 | | 55520408 | | 0.03211 | | 0.888734 | | 1.69E-15 | | 0.00804601 | | 63.39496472 |
| rs56225615 | T | C | 0.0970517 | -0.042291 | | | 0.192771 | | 0.144514 | | 15 | | 79139370 | | 0.042577 | | 0.320572 | | 9.62E-24 | | 0.00966127 | | 100.9108479 |
| rs629301 | T | G | 0.0891313 | -0.065881 | | | 0.785458 | | 0.779356 | | 1 | | 109818306 | | 0.035102 | | 0.0605341 | | 3.62E-21 | | 0.00943867 | | 89.1741292 |
| rs665770 | A | G | 0.0458422 | 0.01095 | | | 0.397902 | | 0.397588 | | 1 | | 201748124 | | 0.030273 | | 0.717581 | | 4.97E-09 | | 0.00783894 | | 34.19922169 |
| rs6750832 | G | A | 0.0594504 | 0.039325 | | | 0.426348 | | 0.399916 | | 2 | | 85758090 | | 0.030721 | | 0.200516 | | 2.03E-14 | | 0.00777244 | | 58.50524546 |
| rs7137258 | A | C | 0.0953264 | -0.090012 | | | 0.0508112 | | 0.055529 | | 12 | | 54512164 | | 0.071316 | | 0.20689 | | 3.52E-08 | | 0.0172895 | | 30.39915533 |
| rs72661887 | T | C | 0.0523715 | -0.00961 | | | 0.537977 | | 0.441441 | | 1 | | 38416310 | | 0.031453 | | 0.759953 | | 1.11E-11 | | 0.00771087 | | 46.12997968 |
| rs72710004 | T | C | 0.109539 | -0.143447 | | | 0.0468209 | | 0.021765 | | 14 | | 100319598 | | 0.13421 | | 0.28515 | | 1.17E-09 | | 0.0180029 | | 37.02138018 |
| rs7412 | T | C | -0.203095 | 0.086356 | | | 0.0533155 | | 0.078147 | | 19 | | 45412079 | | 0.05654 | | 0.126678 | | 3.97E-31 | | 0.0175033 | | 134.6351908 |
| rs754203 | G | A | 0.0461634 | 0.011948 | | | 0.294434 | | 0.303586 | | 14 | | 100157967 | | 0.032598 | | 0.713978 | | 4.28E-08 | | 0.0084259 | | 30.01670205 |
| rs7696431 | G | T | -0.0456509 | -0.032493 | | | 0.44007 | | 0.480074 | | 4 | | 169687725 | | 0.03005 | | 0.279566 | | 4.44E-09 | | 0.0077811 | | 34.42046194 |
| rs9515203 | C | T | -0.0776043 | 0.035676 | | | 0.2616 | | 0.255662 | | 13 | | 111049623 | | 0.037053 | | 0.335629 | | 9.21E-19 | | 0.00877451 | | 78.22144333 |
| rs9852711 | T | C | -0.0792256 | 0.034477 | | | 0.0827672 | | 0.0258 | | 3 | | 152151411 | | 0.098274 | | 0.725718 | | 2.14E-08 | | 0.0141477 | | 31.35879671 |
|  |  |  |  |  | | |  | |  | |  | |  | |  | |  | |  | |  | |  |
| LUCA(exposure) and CAS(outcome) | | | | | | | | | | | | | | | | | | | | | | | |
| SNP | effect_allele | other_allele | beta.exposure | beta.outcome | | | eaf.exposure | | eaf.outcome | | chr | | pos | | se.outcome | | pval.outcome | | pval.exposure | | se.exposure | | F |
| rs11571833 | T | A | 0.472187 | 0.0445586 | | | 0.010904 | | 0.00937416 | | 13 | | 32398489 | | 0.0403714 | | 0.269716 | | 6.12E-16 | | 0.05839 | | 65.39598745 |
| rs11780471 | A | G | -0.141138 | -0.0209692 | | | 0.059619 | | 0.0658083 | | 8 | | 27487202 | | 0.015574 | | 0.178168 | | 1.69E-08 | | 0.025021 | | 31.81841868 |
| rs1629083 | T | C | -0.067017 | -0.00383534 | | | 0.502439 | | 0.472281 | | 11 | | 118255861 | | 0.00771887 | | 0.619274 | | 1.25E-08 | | 0.011772 | | 32.40928567 |
| rs239935 | A | G | -0.066847 | 0.0102783 | | | 0.521537 | | 0.484013 | | 6 | | 166998300 | | 0.0077006 | | 0.181959 | | 1.29E-08 | | 0.011753 | | 32.34941048 |
| rs34720986 | C | G | -0.120016 | 0.00103967 | | | 0.109984 | | 0.123456 | | 6 | | 32625493 | | 0.0116402 | | 0.92883 | | 2.48E-08 | | 0.021529 | | 31.07639126 |
| rs380286 | A | G | -0.141287 | 0.00542497 | | | 0.422631 | | 0.47806 | | 5 | | 1320132 | | 0.00769211 | | 0.480646 | | 1.51E-32 | | 0.011893 | | 141.130724 |
| rs4774488 | C | T | 0.06581 | 0.00802458 | | | 0.404215 | | 0.329726 | | 15 | | 47199094 | | 0.00818873 | | 0.327109 | | 3.60E-08 | | 0.011945 | | 30.35368853 |
| rs55781567 | G | C | 0.260379 | -0.0153639 | | | 0.366674 | | 0.334415 | | 15 | | 78565644 | | 0.00815241 | | 0.0594853 | | 3.08E-103 | | 0.012068 | | 465.5231753 |
| rs71658797 | A | T | 0.127764 | 0.00523966 | | | 0.103378 | | 0.129313 | | 1 | | 77501822 | | 0.0115229 | | 0.649313 | | 3.25E-11 | | 0.019257 | | 44.01895613 |
| rs7705526 | A | C | 0.117126 | -0.016112 | | | 0.340315 | | 0.323868 | | 5 | | 1285859 | | 0.00824077 | | 0.0505638 | | 1.01E-18 | | 0.013259 | | 78.03421798 |
| rs77468143 | G | T | -0.082663 | 0.0184182 | | | 0.253876 | | 0.293443 | | 15 | | 49084427 | | 0.00846505 | | 0.0295706 | | 1.00E-09 | | 0.013532 | | 37.31628033 |
| rs7953330 | C | G | -0.087266 | 0.0024262 | | | 0.312094 | | 0.363421 | | 12 | | 889653 | | 0.00800594 | | 0.761853 | | 6.10E-12 | | 0.012689 | | 47.29719058 |
|  |  |  |  |  | | |  | |  | |  | |  | |  | |  | |  | |  | |  |
| LUSC (exposure) and CAS (outcome) | | | | | | | | | | | | | | | | | | | | | | | |
| SNP | effect_allele | other_allele | beta.exposure | beta.outcome | | | eaf.exposure | | eaf.outcome | | chr | | pos | | se.outcome | | pval.outcome | | pval.exposure | | se.exposure | | F |
| rs11571818 | C | T | 0.753896 | 0.0531454 | | | 0.010732 | | 0.00975684 | | 13 | | 32394673 | | 0.0396254 | | 0.179857 | | 9.77E-16 | | 0.093889 | | 64.47531182 |
| rs467095 | C | T | -0.178831 | 0.00428373 | | | 0.426949 | | 0.481447 | | 5 | | 1336106 | | 0.00768732 | | 0.577359 | | 6.73E-21 | | 0.019069 | | 87.94877053 |
| rs7953330 | C | G | -0.145613 | 0.0024262 | | | 0.315268 | | 0.363421 | | 12 | | 889653 | | 0.00800594 | | 0.761853 | | 7.26E-13 | | 0.020296 | | 51.4729893 |
| rs8040868 | C | T | 0.254984 | -0.0191768 | | | 0.413519 | | 0.378668 | | 15 | | 78618839 | | 0.00791103 | | 0.0153483 | | 2.50E-41 | | 0.018936 | | 181.3215278 |
