# Supplementary material for: Coronary atherosclerosis has a protective genetic causal effect against lung squamous cell carcinoma: A bidirectional two-sample Mendelian randomization study based on STROBE-MR guidelines
Source: Medicine (Baltimore). 2025 Jul 25;104(30):e43378. doi: 10.1097/MD.0000000000043378 (PMC12303480; doi:10.1097/MD.0000000000043378)
Supplement: Supplementary file 3 [file medi-104-e43378-s003.docx]

**Title**: Coronary atherosclerosis has a protective genetic causal effect against lung squamous cell carcinoma: a bidirectional two-sample Mendelian randomization study.

**First author**: Zhicheng liao

**Supplementary material 3: Funnel plots, Scatter plots and Leave-one-out plots**

**Supplementary Figure 1:** **Funnel plots illustrate the symmetrical distribution of individual variant estimates around the point estimate.**

| 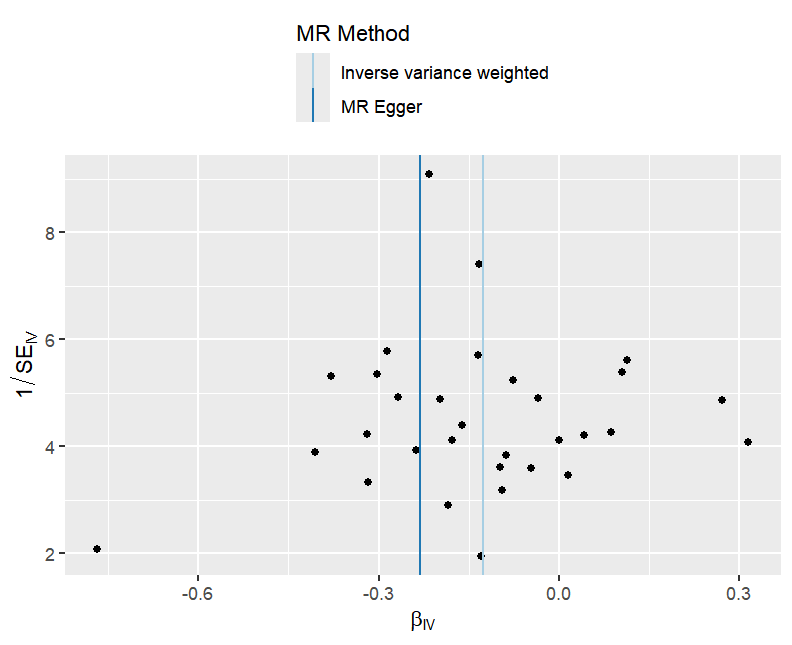  (A) | 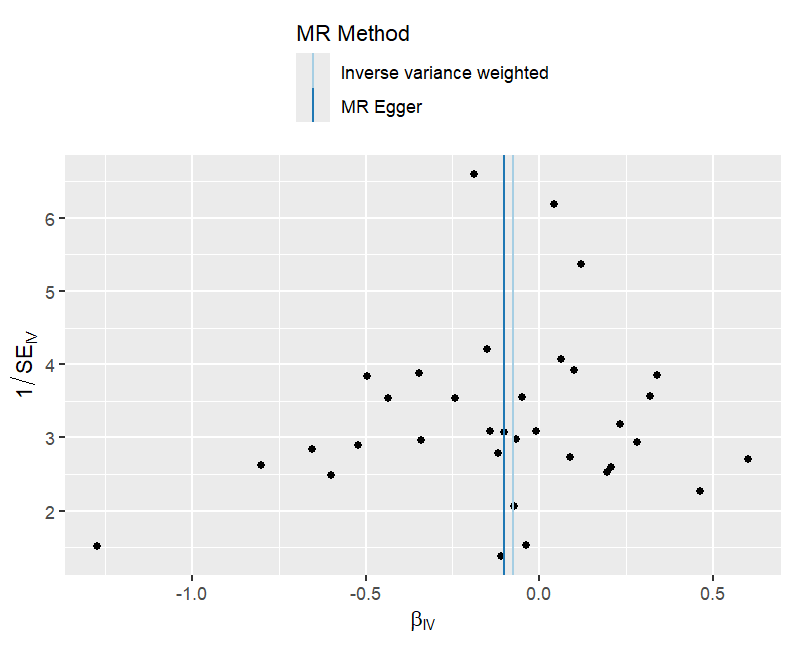  (B) | 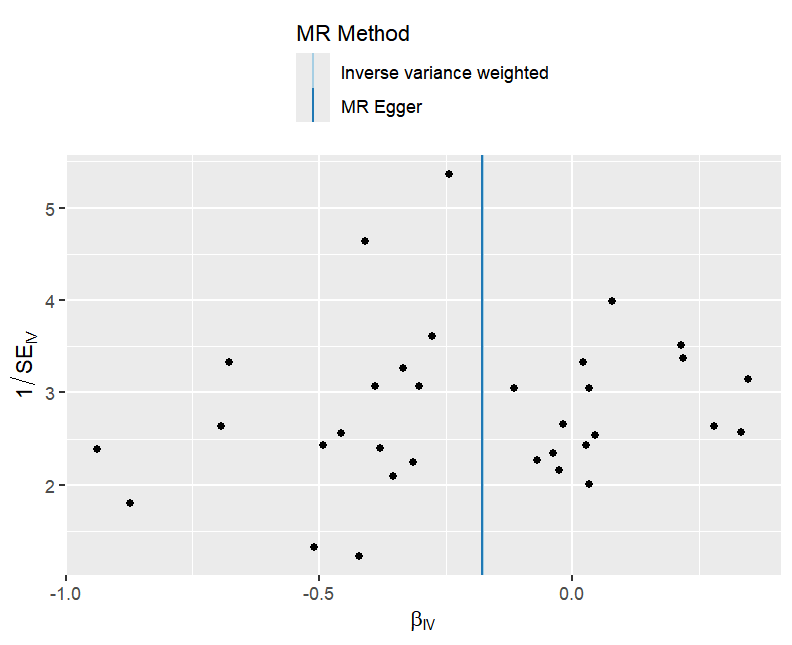  (C) |
| --- | --- | --- |
| 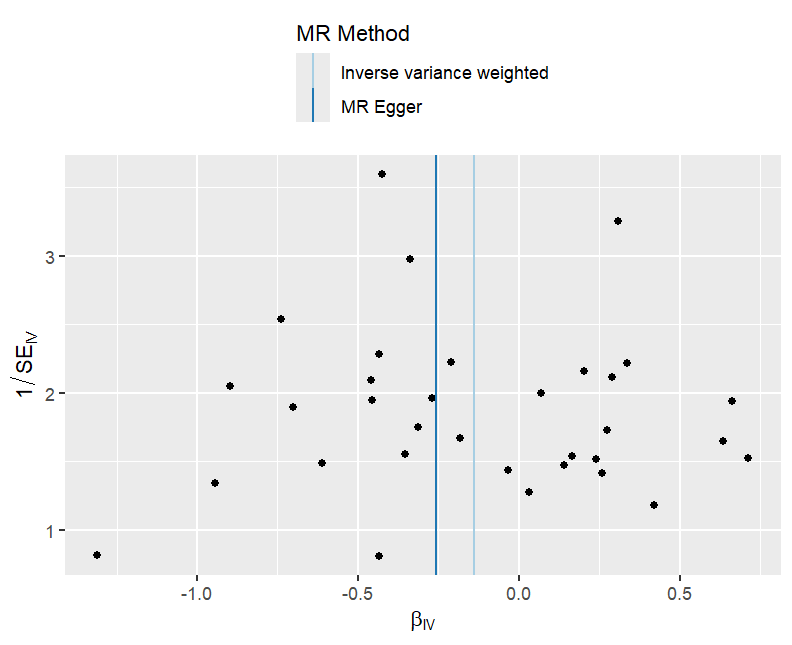  (D) | 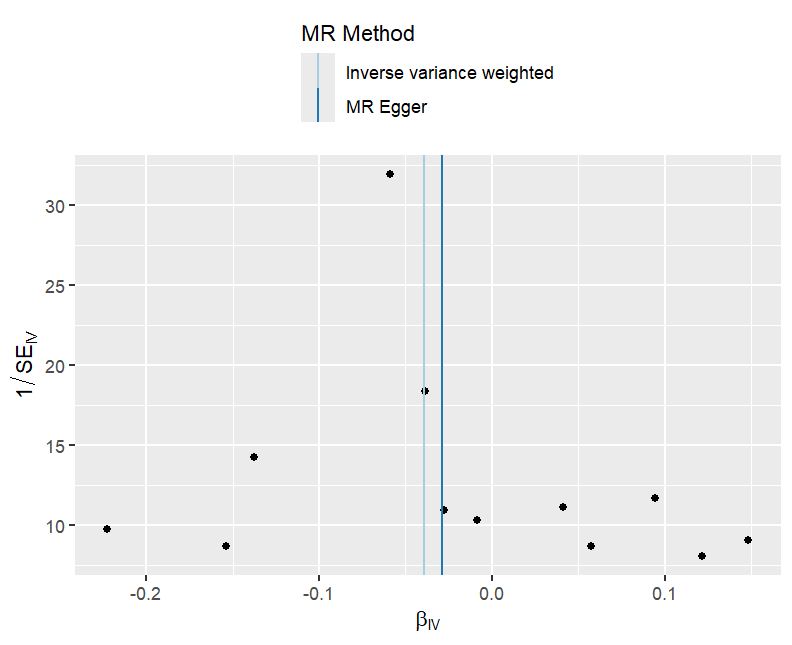  (E) | 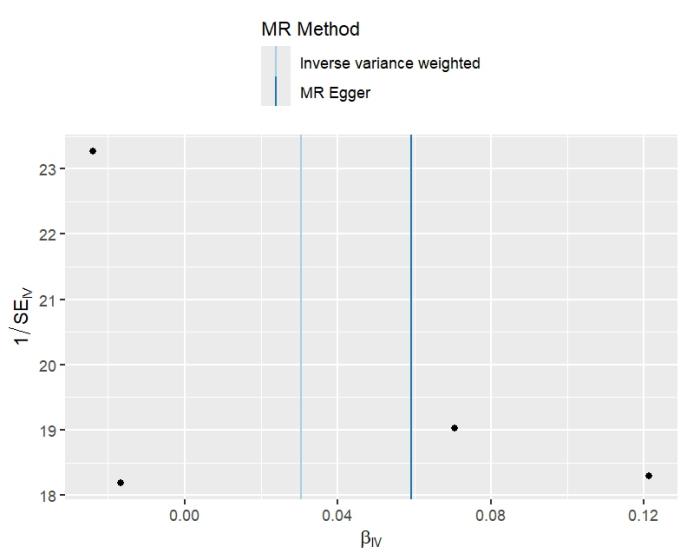  (F) |

Figures (A-D) represent the relationships between CAS as an exposure factor and LUCA, LUAD, LUSC, and SCLC as outcome variables, respectively. Figures (E) represents the relationship between LUCA as an exposure factor and CAS as an outcome variable. Figures (F) represents the relationship between LUSC as an exposure factor and CAS as an outcome variable. The x-axis represents the Mendelian randomization estimate of individual SNPs, while the y-axis represents the inverse of their standard error.

**Supplementary Figure 2:** **Scatter plots illustrate the influence of SNPs on CAS to LUCA and its subtypes.**

| 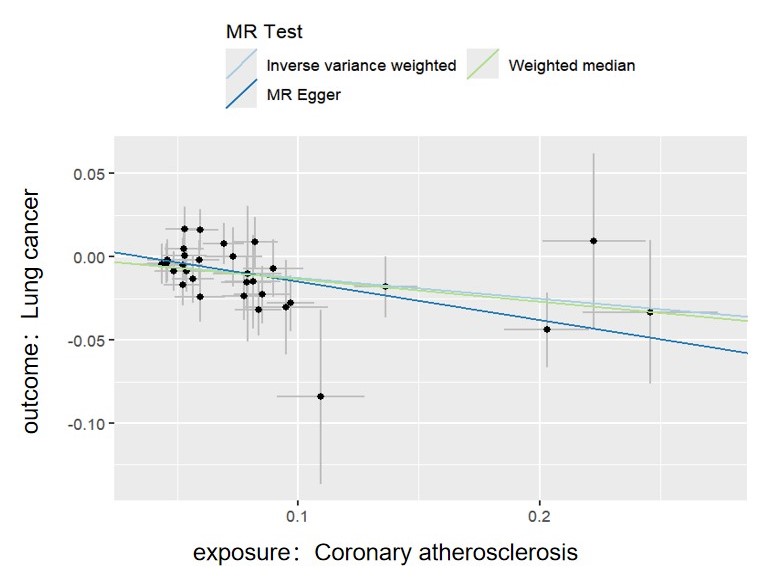(A) | **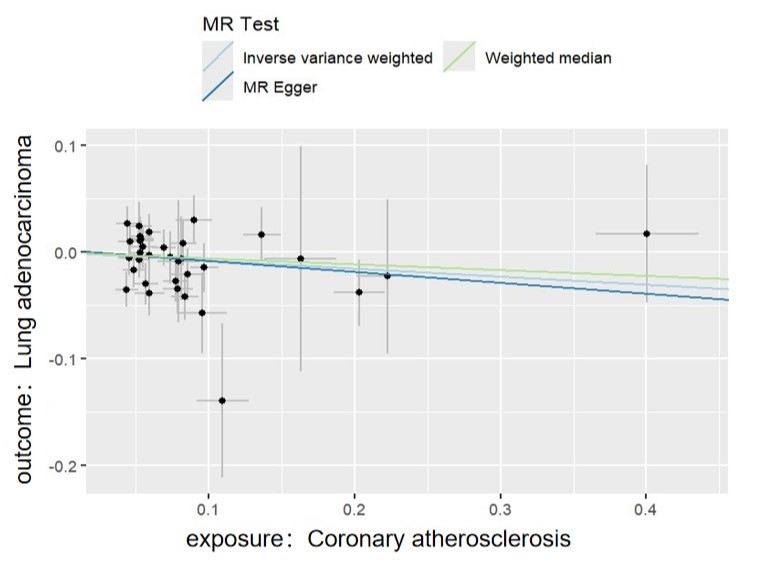**(B) | **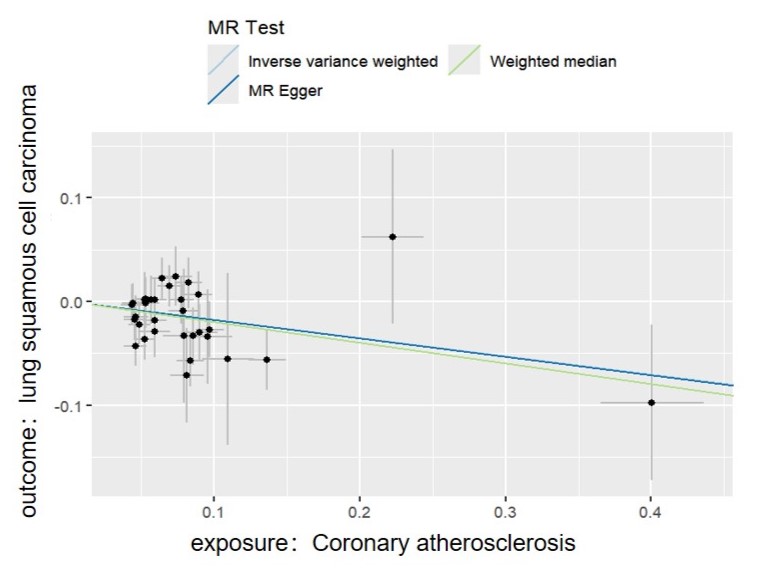**(C) |
| --- | --- | --- |
| **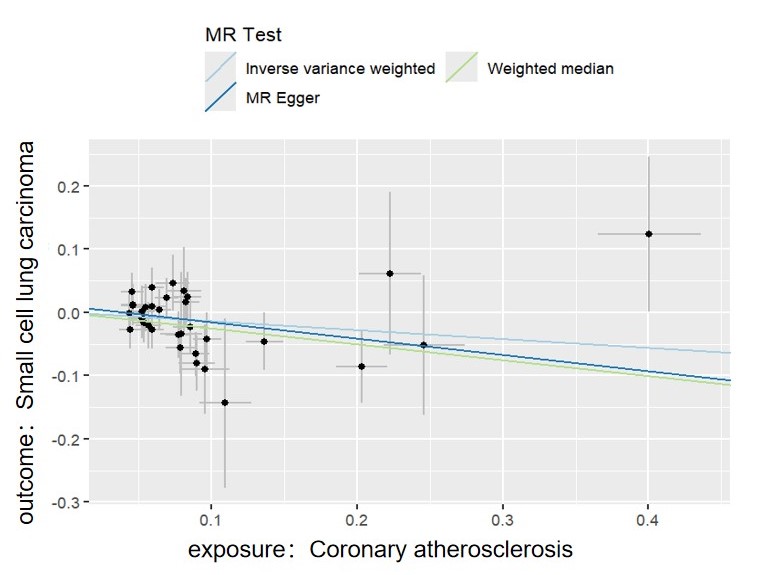**(D) | 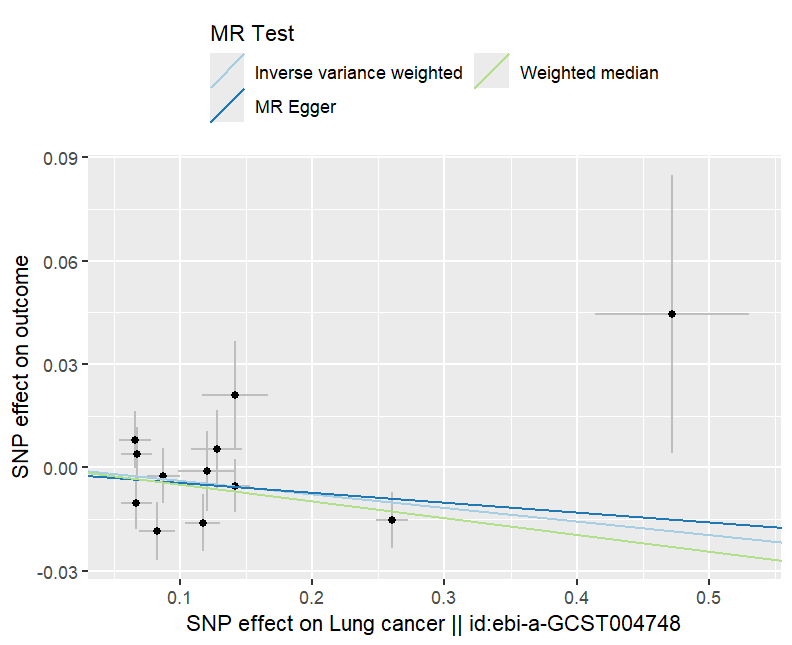(E) | **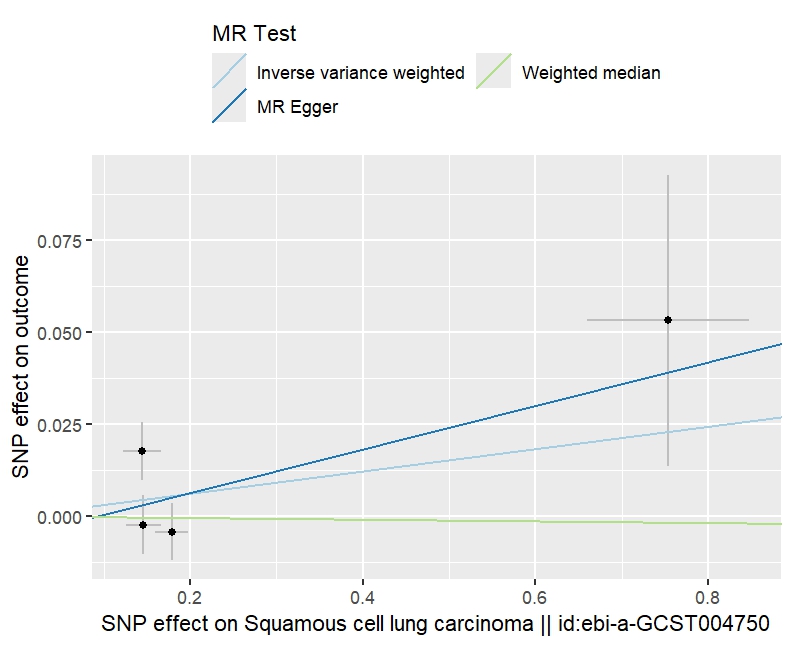**(F) |

Figures (A-D) represent the relationships between CAS as an exposure factor and LUCA, LUAD, LUSC, and SCLC as outcome variables, respectively. Figures (E) represents the relationship between LUCA as an exposure factor and CAS as an outcome variable. Figures (F) represents the relationship between LUSC as an exposure factor and CAS as an outcome variable. The colors of the fitted line correspond to the three approaches used in univariable Mendelian randomization (MR) analyses.

**Supplementary Figure 3: The leave-one-out method reflects the stability of the results of Mendelian randomization analysis.**

| 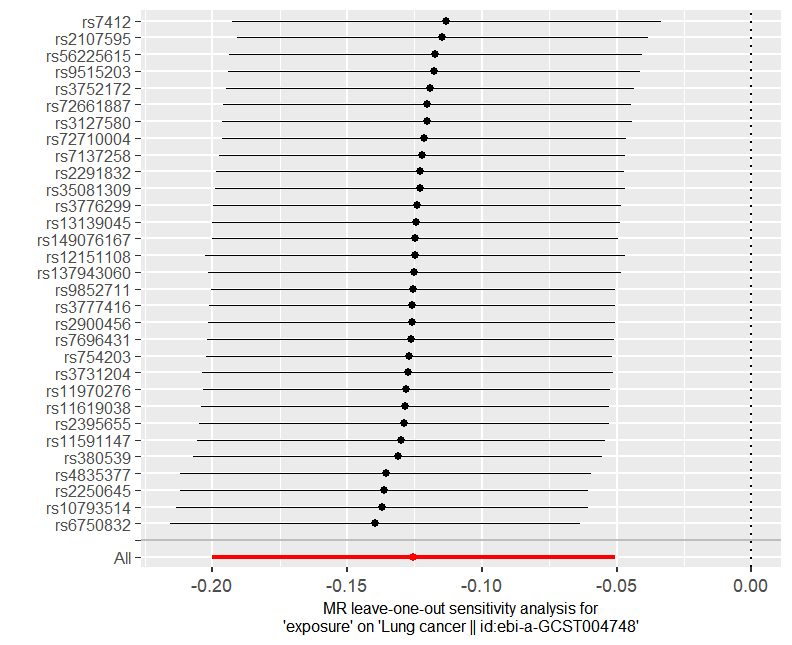  (A) | 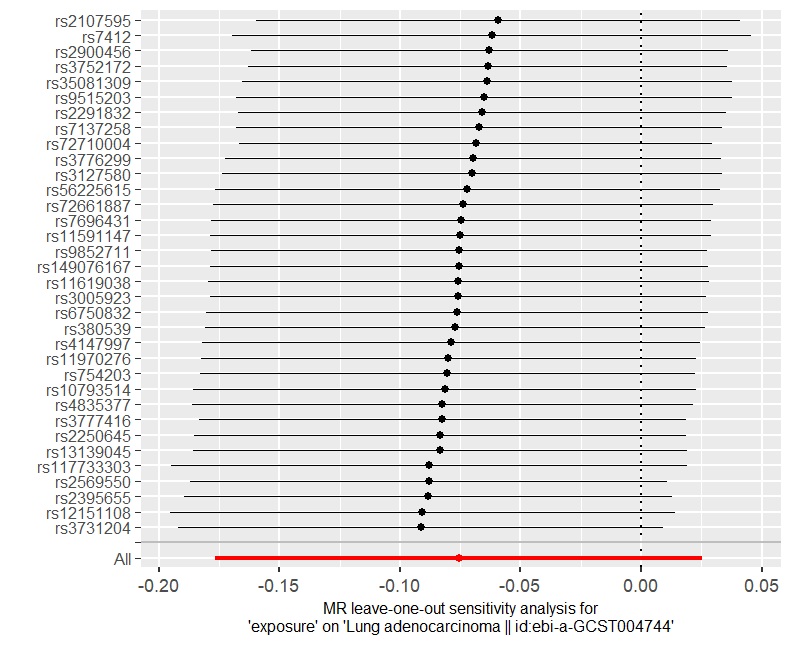  (B) | 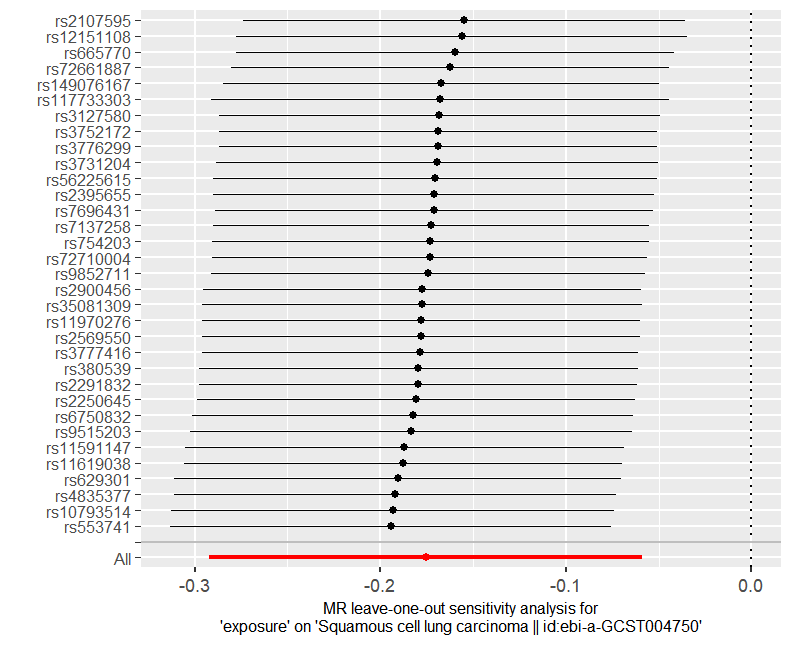  (C) |
| --- | --- | --- |
| 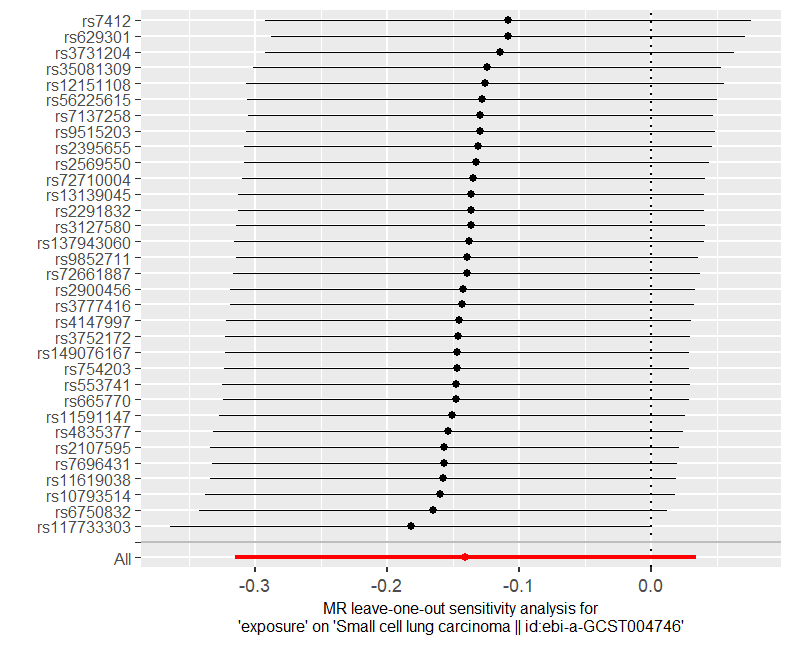  (D) | 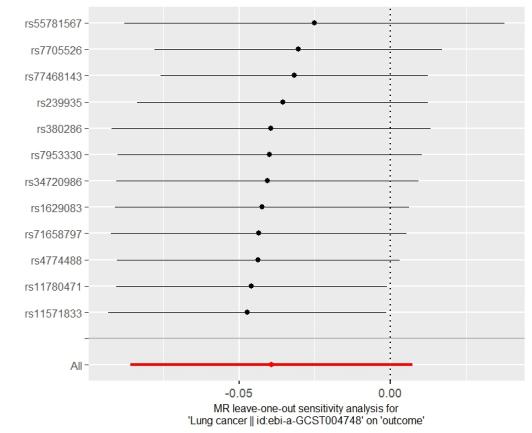  (E) | 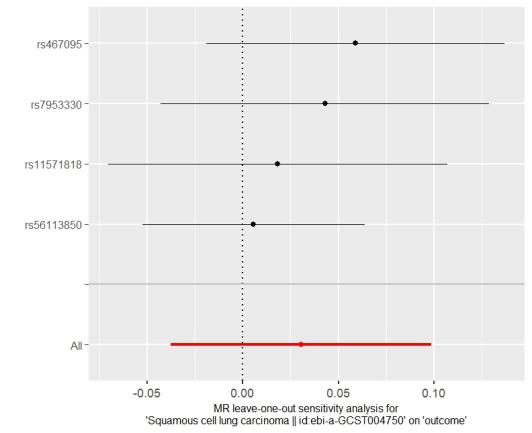  (F) |

Figures (A-D) represent the relationships between CAS as an exposure factor and LUCA, LUAD, LUSC, and SCLC as outcome variables, respectively. Figures (E) represents the relationship between LUCA as an exposure factor and CAS as an outcome variable. Figures (F) represents the relationship between LUSC as an exposure factor and CAS as an outcome variable. The red line represents the estimated causal effect calculated using all SNPs, reflecting the average causal relationship between the overall genetic association and exposure and outcome. The black line illustrates the trajectory of estimated causal effects obtained by analyzing the remaining SNPs after each SNP is individually removed. The dotted line is employed to mark the 95% confidence interval boundary of the point estimate, illustrating the range of uncertainty.
